# Letters, Words, Sentences, and Reading

**DOI:** 10.5334/joc.396

**Published:** 2024-08-27

**Authors:** Jonathan Grainger

**Affiliations:** 1 Centre de Recherche en Psychologie et Neurosciences, CNRS & Aix-Marseille University, Marseille, France; 2 Institute of Language, Communication, and the Brain, France

**Keywords:** Reading, Letter identification, Word recognition, Sentence processing

## Abstract

In this personal, and therefore highly selective, review article I summarize work performed in collaboration with numerous colleagues on how skilled adult readers perform identification tasks and speeded binary decision tasks involving single letters and visually presented words and sentences. The overarching aim is to highlight similarities in the processing performed at three key levels involved in written language comprehension (in languages that use an alphabetic script): letters, words, and sentences. The comparisons are made using behavioral data obtained with: i) speeded (response-limited) binary decision tasks; and ii) the effects of simultaneous surrounding context on letter and word identification using both data-limited (non-speeded) and response-limited procedures. I then propose a general framework that combines the three levels of processing, and that connects core processes at each level with the processing involved in tasks designed to reflect those core processes, and I end by suggesting possible avenues for future research with an aim to extend this general framework.^1^

## 1. Letter identification

Isolated letter identification has received relatively little attention from experimental psychologists compared with research on visual word recognition. Yet, as noted by Hofstadter (1985), the crux of intelligence, whether it be human or artificial, lies in the knowledge of what constitutes the letter “A” (A = a ≠ e). Furthermore, given the overwhelming evidence that reading words in languages that use an alphabetic script is essentially letter-based (i.e., words are recognized via their component letters), this implies that in order to fully understand visual word recognition (and from there, sentence and text reading), one must first understand how abstract letter identities are derived from the various visual forms that a given letter can adopt. In sum, it is clear from the evidence at present that skilled readers do not use sub-letter features as the principal means to identify written words, and even less so to understand written sentences. Therefore, the starting point of understanding skilled reading in languages that use an alphabetic orthography is indeed understanding the processes involved in isolated letter identification.^2^

In prior explorations of single letter identification, one oft-used task is speeded (response-limited) letter naming (e.g., Bowers et al., 1998). Unlike word naming, where the naming response can be initiated prior to identification of the whole word, letter naming necessarily involves recovery of the letter identity and its associated name, and therefore should in principle be a good reflection of the time it takes to identify a letter. Nevertheless, letter naming does involve an articulatory component that likely causes variation in response times (RTs) over and above the time it takes to identify a letter (e.g., Ziegler et al., 2000; but see Madec et al., 2012, for a possible remedy). In my own work I have predominantly used two other behavioral tasks to study basic processes in letter identification: one response-limited (the alphabetic decision task – where participants have to classify stimuli as being a letter or not as rapidly and as accurately as possible), and one data-limited (the perceptual identification task – where participants have to identify a briefly presented letter with no time pressure). Although performance in the alphabetic decision task does involve a decision component that operates over and above the process of letter identification, performance in this task has been shown to be more similar to perceptual identification than letter naming (Grainger et al., 2008). Moreover, the advantage of alphabetic decision compared with perceptual identification is that data (RTs and accuracy) are obtained on every trial. The key for rendering the alphabetic decision task a good measure of letter identification time is the nature of the pseudo-letter stimuli that are used as foils. Great progress has been made here with the creation of artificial pseudo-letter fonts such as the BACS stimulus set (Vidal et al., 2017). The use of well-designed pseudo-letters in the alphabetic decision task forces the observer to identify a letter before triggering a response, rather than the response being triggered by some form of letter-likeness that would be greater for letter stimuli than pseudo-letter stimuli.

Another behavioral task that has been used to study letter identification is the same-different matching task, where participants are asked to decide if two simultaneously or sequentially presented letters are the same or not. Data-limited and response-limited versions of this task have been used, as well as a combination of the two. This task has been widely used to uncover the visual features involved in letter identification in different writing systems, such as the work with the Roman alphabet (e.g., Courrieu et al., 2004; Simpson et al., 2013) and work with the Arabic alphabet (e.g., Boudelaa et al., 2020; Wiley et al., 2016). The response-limited version of this task (Courrieu et al., 2004) has recently been applied to provide a measure of visual similarity between pairs of words by using a combined measure of the visual similarities of their component letters (Massol & Grainger, 2024).

Another means to provide more leverage with respect to uncovering basic processes in letter identification is to add a priming manipulation (e.g., Jacobs & Grainger, 1991). This has been done with the alphabetic decision task and the letter naming task. Manipulations of the nature of the overlap (nominal – e.g., e–E vs. visual – e.g., F–E) between non-identical prime and target letter stimuli, plus a manipulation of prime duration (Jacobs et al., 1995; Ziegler et al., 2000), have revealed that visual overlap is the main factor determining the size of priming effects in the alphabetic decision task, whereas nominal identity (i.e., visually dissimilar versions of the same letter such as a-A vs. e-A) is the main factor determining priming effects in letter naming. This underlines the importance of interpreting behavioral effects as a function of the task used to measure such effects, and most notably the difference between tasks that involve articulation or not.

A further means to provide increased leverage with respect to uncovering basic processes in letter identification is to combine behavioral tasks with electroencephalography (EEG). EEG recordings provide invaluable information with respect to the timing of effects seen in the behavioral data. This is crucial when testing models that have such a temporal component. Examples of such studies that I have co-authored include Petit et al. (2006), Rey et al. (2009), Madec et al. (2012), and Winsler et al. (2022). Interested readers are referred to these individual papers for more details, but, in sum, these studies confirm the classic picture (already painted by Selfridge, 1988, and revisited in Grainger et al., 2008) whereby hierarchically arranged visual features of varying complexity converge on letter-level representations which are initially case-specific before achieving abstract, case-independent letter identification.

In the following section I will show how this basic module for single letter identification can then be extended to a network capable of identifying multiple letters at a time by aligning a bank of letter detectors along the horizontal meridian (for written languages that use horizontally aligned alphabetic scripts). The general idea is that this horizontally aligned bank of letter detectors represents the first stage of orthographic processing following lower-level visual processing (although I have proposed that letters can be considered to be complex visual features: Massol & Grainger, 2022). Although not yet specified in published work, I propose, following the work on individual letter identification briefly mentioned above, that this very first level of orthographic processing is case-specific. That is, a letter is first identified as an uppercase or lowercase version of the same letter identity. This case information is then ignored in higher-level orthographic processing (leading to word recognition in written languages such as English and French), but it is not completely lost, since this information remains available for higher-level processing of word sequences (indicating the beginning of a sentence for example) or special cases of orthographic forms (e.g., brand names, logotypes: see e.g., Labusch et al., 2024). Case information also contributes to distinguishing between common and proper nouns, and between nouns and other parts of speech in German, hence providing a further facilitatory input to the process of skilled reading (see e.g., Labusch, Kotz, et al., 2022; Peressotti, et al., 2003; Sulpizio & Job, 2018).

## 2. The letter-word interface

My own research on letter identification and word recognition (Grainger & Jacobs, 1994; 2005) was heavily influenced by one particular phenomenon, and one particular model. The phenomenon is the “word superiority effect” (Cattell, 1886; Reicher, 1969; Wheeler, 1970), and the model is the “Interactive-Activation model” (McClelland & Rumelhart, 1981; Rumelhart & McClelland, 1982) which was designed to account for the word superiority effect. The word superiority effect refers to the greater ease with which individual letters are identified when presented in the context of an existing word compared with nonwords. Conclusive evidence for this was provided by two independent articles using a paradigm – the Reicher-Wheeler task (Reicher, 1969; Wheeler, 1970) – designed to rule-out sophisticated guessing accounts of why letters are better identified in words compared with nonwords. That is, given the data-limited rather than response-limited procedure participants could use partial information derived from the stimulus (e.g., T??LE) to guess that the letter at position 3 is the letter B.^3^

The word superiority effect spans the major part of the history of Experimental Psychology, being first observed by Cattell (1886) in Wundt’s laboratory in Leipzig.^4^ Cattell’s observation that words were processed just as efficiently as single letters led him to question the role played by single letters in word recognition. Cattell questioned how a word could be recognized via its component letters if these individual letters are just as hard to identify as the whole word? In Grainger and Hannagan (2014) we referred to this interrogation as “Cattel’s conundrum”. However, Cattell’s observation involved a comparison of single letter identification and whole-word identification, and it has since been reported that the results of such a comparison depend on the nature of the masking procedure used in the perceptual identification task (Jordan & De Bruijn, 1993). Crucially, Jordan and De Bruijn demonstrated that when the masking conditions were comparable for the single letter and word conditions, then there was no significant difference between the two.

When three conditions (words, pseudowords, nonwords) are tested in the Reicher-Wheeler paradigm or simple post-cued letter-in-string identification, then one observes that the “word superiority effect” (i.e., word vs. pseudoword) is in fact quite small in magnitude compared with the “pseudoword superiority effect” (i.e., pseudoword vs. nonword: e.g., Grainger et al., 2003). That is identification accuracy of the letter “B” at the 3^rd^ position in the word “TABLE” is significantly higher than identification of the same letter at the same position in the pseudoword “PABLE”, but this difference is much smaller compared to the comparison between “PABLE” and “PFBGT”. This pattern points to orthographic and/or phonological statistical regularities (often referred to as phonotactic constraints) as a major factor in determining performance in the Reicher-Wheeler task, rather than stimulus lexicality per se. That is, the fact that the letter B is more often encountered in the context of ABL than FBG would the primary source of the “word” superiority effect. Nevertheless, one study has shown that word mis-identification also plays a role (Grainger & Jacobs, 2005). The idea is that when presented with the pseudoword PABLE some participants on some trials will actually perceive the word TABLE and will therefore be able to infer that B is the central letter. Such slow inferential processes are possible in data-limited paradigms such as the Reicher-Wheeler task, and even the careful design of experiments using the Reicher-Wheeler task does not rule out this possibility (i.e., if upon presentation of the word TABLE you are able identify that word, then, when tested with T vs. C at the first position you will respond “T”).

Crucially, all the above is only possible under the assumption of parallel letter processing as in the Interactive-Activation model, unless one were to propose a very fast serial scan mechanism (e.g., Whitney, 2001). However, the data at present fit better within a parallel processing framework in which all letters are processed in parallel within the limits of visual acuity (i.e., imposing a limit on word length whereby longer words must be broken down into smaller size units such as morphemes) and this parallel letter processing would be modulated by other factors – most notably crowding and spatial attention (Grainger et al., 2016; see Adelman et al., 2010, for evidence for parallel letter processing).

So, summing-up the first two sections that focused on single letter processing, the evidence at present points to the rapid extraction of abstract letter identities (i.e., independent of case, size, and with a certain degree of tolerance to shape distortion: e.g., Hannagan et al., 2012). These abstract letter identities are thought to form the very first building blocks of the reading process enabling the rapid identification of orthographic word forms via parallel letter processing. This transition from letters to words is performed by a cascaded-interactive processing network such that early activation of orthographic word identities can feedback information to the on-going process of letter identification, hence facilitating this process and rendering the overall process of word identification more robust (i.e., tolerant to noise). In section 4 I will show how these general principles also apply to word-sentence interactions, but before that we need to examine the basics of visual word recognition.

## 3. Orthographic processing and word recognition

Words are undeniably the building blocks of reading (the equivalent of the cell for biology according to Balota et al., 2006) and language comprehension in general (although morphemes may play a greater role in highly agglutinative languages such as Finnish, and morpheme-based written languages such as Chinese). In all western scripts, written words are delimited by spaces, but this was not always the case. Latin, for example, was originally written without spaces between words. According to Saenger (1997) the introduction of between-word spaces corresponds to the shift from reading aloud to silent reading. The spacing between words that was introduced into many occidental scripts renders individual words more salient and reinforces their role as the building blocks of the overall process of reading comprehension.

When reading a text, after performing a preliminary analysis of the letters that compose that text, readers then identify words and put them in order with the aim to achieve a meaningful interpretation of the sequence of squiggles on the page they are reading. In this section I will summarize research on single word recognition before describing research investigating the impact of surrounding context on the word recognition process in the following section.

One task has dominated research on single word recognition – this is the lexical decision task – decide as rapidly and as accurately as possible if a sequence of letters is a real word or not. Word naming was also an important task used to investigate single word recognition, but the influence of articulatory factors on performance in this task led to its relegation as a task useful for understanding the conversion of orthographic information to phonological information which is indeed an essential ingredient of the process of reading aloud (see Coltheart et al., 2001; and Seidenberg and McClelland, 1989, for computational models of word naming). The lexical decision task was initially used to investigate the impact of intrinsic word properties (e.g., word length, word frequency) on visual word recognition. It was then extended to investigate the impact of “virtual” variables, such as a word’s orthographic neighborhood (e.g., Andrews, 1989; Grainger et al., 1989; Grainger, 1990). It was also the main task used to demonstrate the influence of phonological variables on silent word reading (i.e., when the task does not require overt phonological/articulatory processing: e.g., Ferrand & Grainger, 1992, 1994; see Frost, 1998, for a review).

Apart from the investigation of intrinsic and virtual variables associated with individual words, there are two other main ways in which the lexical decision task has been used to uncover the basic processes involved in single word reading. These are: 1) the combination of masked priming with lexical decision (Forster & Davis, 1984), and 2) the manipulation of the nature of the nonword stimuli in the task with a focus on how these nonwords are processed. I will focus on two key phenomena observed with these versions of the lexical decision task: transposed-letter effects and repeated-letter effects.

The transposed-letter effect, first reported by Bruner and O’Dowd (1958), was to become one of the most important phenomena motivating novel accounts of orthographic processing in general, and letter position coding in particular (see Grainger, 2008, for a review). The importance of this phenomenon was not only scientific, but also in terms of attracting attention to fundamental scientific issues in skilled reading research via various forms of media, starting with the so-called “Cambridge University email”. To this day, transposed-letter effects remain an eye-catcher for the general public. But what do they reflect? How do reading researchers interpret this phenomenon? Transposed-letter effects, observed with various paradigms, were responsible for the development of two key approaches to letter-position coding during visual word recognition: one focusing on the role of positional uncertainty (e.g., Gomez et al., 2008), and the other focusing on flexibility in letter-order encoding (e.g., Grainger & van Heuven, 2004).

When examining the essence of these two approaches to letter position coding, one major difference between the two lies in the mechanism used to encode for the order of letters in a word – a key ingredient of accurate word recognition given the number of anagrams that exist in alphabetic languages (Grainger & Hannagan, 2014). The positional noise or uncertainty approach (e.g., Gomez et al., 2008) posits that upon presentation of a written word such as TABLE, an ordinal representation of its component letters is created (i.e., T = 1, A = 2, B = 3, L = 4, E = 5). Just exactly how this is achieved is not specified. Crucially, positional noise is added to this ordinal representation such that evidence that there is a B at position 3 spreads to neighboring positions leading to the possibility that the B might be at position 2, for example. The flexible letter-order encoding account (Grainger & van Heuven, 2004; Whitney, 2001), on the other hand, posits two distinct levels of how letter position information is represented. First, letters are associated with a specific location in a string of letters, but at this level letter-order information has not yet been encoded. Letter-order is inferred from the information provided by the first level of processing. In Whitney’s model this is achieved using the information provided by a fast serial scan of the letter string. In Grainger & van Heuven’s model this is achieved by inferring relative letter positions from information about where a given letter is relative to eye fixation location (e.g., if the T is further to the left of fixation than the letter B then the T must be before the B).^5^

**Figure 1:**
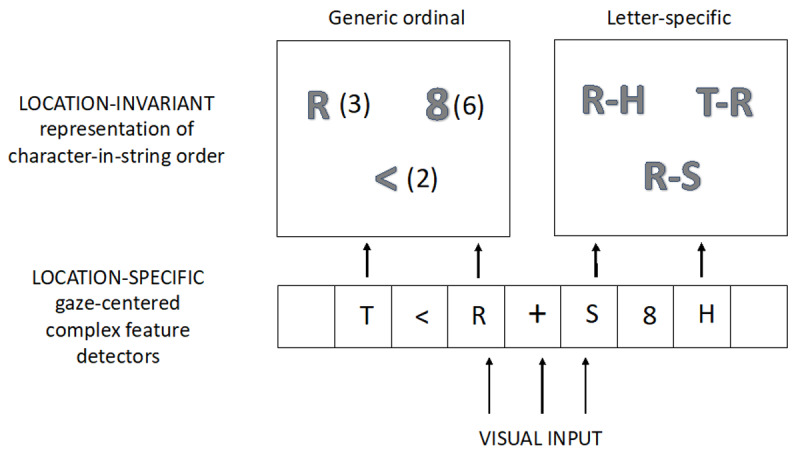
From Massol and Grainger (2022). Stimuli are first encoded via location-specific gaze-centered complex feature detectors. Location-specific complex features then activate location-invariant object identities that are assigned an order in the string. Crucial is the hypothesized distinction between two different location-invariant order-encoding mechanisms: a generic order encoding mechanism, illustrated here by a simple ordinal representation (e.g., R(3), there is the letter R at the 3rd position), and a letter-specific order encoding mechanism – open-bigram coding (e.g., R-H, there is an R before an H, possibly with other letters/characters in between). Note that although the component letters of open-bigrams are ordered, the bigrams themselves are not.

Evidence in favor of the two-step (location-then-order) encoding model has been provided by comparing transposed-character effects obtained for letters vs. other kinds of characters (digits, symbols) in the same-different matching task (Duñabeitia et al., 2012; Massol et al., 2013; Massol & Grainger, 2022; 2024).^6^ The key argument here is that although different types of character might well be processed the same way at the level of location-specific processing (via complex feature detectors, Massol & Grainger, 2022), the flexible order-encoding mechanism should only operate for letter sequences, where a certain amount of flexibility can enable fast but accurate word recognition. This is clearly not the case for number processing where the exact location of each digit in the multi-digit number is crucial for the computation of the numerical value of the ensemble. The results at present clearly point to a letter-specific mechanism for order-encoding that gives rise to greater transposed-letter effects compared with transposed-effects obtained with other types of character (see Grainger & Hannagan, 2014, for a review). Couched within the general approach for orthographic processing proposed by Grainger and van Heuven (2004), this has resulted in the more general model of location and order encoding for strings of characters presented in Massol and Grainger (2022) and shown in Figure 1.

A much less studied, but equally important phenomenon concerns the effects of letter repetition on orthographic processing. I became interested in letter repetition effects upon noticing that when a word that I had typed and thought I had correctly spelled was underlined in red (MS word’s way of indicating misspellings), it was often because I had failed to notice a letter repetition (e.g., silencne). Although I had previously studied such letter repetition effects on word recognition (Schoonbaert & Grainger, 2004), it was the work of Trifonova and Adelman (2019) that regenerated my interest in this largely under-studied phenomenon. In a re-analysis of mega-study word recognition data, Trifonova and Adelman found that the presence of a letter repetition in words made lexical decisions to these words harder (thus replicating the results reported by Schoonbaert & Grainger, 2004). This, however, was only the case for non-adjacent letter repetitions, which is in line with the evidence for double-letters being a special case (e.g., Fischer-Baum, 2017).

This pattern of letter repetition effects is exactly what open-bigram coding predicts. So, we ran a further letter repetition study (Kerr et al., 2021), using the paradigm for studying transposed-letter effects introduced by Bruner and O’Dowd (1958). We found that nonwords created by inserting a letter already present in a baseword (e.g., silecnce from “silence”), were harder to classify as nonwords in a lexical decision task relative to nonwords created by inserting a letter that was not already present in the baseword (e.g., silernce). One important result from the Kerr et al. study is that when both RT and error rates are taken into consideration by using a combined measure referred to as inverse efficiency (mean RT divided by probability correct per condition and participant) then the effect of letter repetition diminishes linearly as a function of the distance between the repeated letters (excluding the case of adjacent letters, which were not tested). The key question now is how well different models of orthographic processing (e.g., open-bigram, overlap) can account for this pattern of effects, and what these models predict in terms of novel manipulations of letter repetitions. Open-bigram coding (Grainger & van Heuven, 2004; Whitney, 2001) accounts for letter repetition effects by the fact that letter repetitions reduce the number of distinct bigrams that are involved in recognizing a given word (or deciding that a nonword is indeed a nonword). The overlap model (Gomez et al., 2008) accounts for letter repetition effects by weighting repeated letters as a single letter as a function of the distance between these letters (see Kerr et al., 2021, for a description of the results of simulations provided by Pablo Gomez).

Furthermore, it remains to be seen if a similar pattern can be observed with letter omission effects by comparing lexical decision times to nonwords formed by removing a repeated vs. a non-repeated letter in a word (e.g., comparing “balnce” vs. “balace” derived from the baseword “balance”). Although Schoonbaert and Grainger (2004) found no effect of this factor in a masked priming experiment (where the manipulation was on the prime stimuli), it remains to be seen if effects might emerge in a simple (unprimed) lexical decision experiment. Open-bigram theory predicts that “nonword” decisions should be harder to make when the omitted letter is a repeated letter. So, it should be harder to decide that “balnce” is not a word compared with “balace” while controlling for effects of orthographic and phonological similarity with real words that could arise with a simpler ordinal encoding of letter and phoneme order.

## 4. Sentence processing and word-sentence interactions

Much behavioral research on sentence reading has been dominated by eye-movement measures (i.e., the movements of the eyes during reading – saccades and re-fixations – and the dwell times when they are stationary – fixation durations). This line of research has had a major impact on theories of skilled reading (see Rayner, 1998, for review), leading notably to the highly influential EZ-Reader model (Reichle et al., 1998). Nevertheless, and without being disrespectful to this extremely important sub-domain of reading research, one must keep in mind that eye-movement measures of reading behavior reflect the reader’s decision concerning when and where to move their eyes to hypothetically optimize the extraction of meaning from print when reading a meaningful text (e.g., the “optimal viewing hypothesis”: O’Regan & Jacobs, 1992). However, there are studies that suggest that eye-movement measures are not a particularly good measure of reading-for-meaning since similar patterns of eye-movements have been observed when scanning meaningless text – so-called “mindless reading” (e.g., Vitu et al., 1995). My own position lies somewhere between these two extremes, with the more practical goal of obtaining converging measures from different paradigms. That is, eye-movement measures certainly do reflect, to a certain extent, the mechanics of skilled reading, including the influence of cognitive factors, but they are not necessarily a perfect measure.

Within this general perspective and given that most theoretical developments in the field of single word recognition have been based on the results obtained with measures other than eye-movement recordings, it appeared logical that alternative measures of sentence comprehension were necessary for the field to advance. Here I will describe three paradigms, or variants of paradigms, that were introduced in order to fill this gap. I will also describe how these paradigms provide a sentence-level analog to pre-existing tasks designed to study visual word recognition. The overall endeavor, as will be seen in section 5, is to connect paradigms and results obtained at the various levels involved in skilled reading.

Two of these paradigms involved the investigation of the effects of surrounding context on single word recognition using: 1) the flankers task; and 2) post-cued word-in-sequence identification. In a daring move forward in terms of paradigm shifts in reading research, Dare and Shillcock (2013) adapted the classic flankers task (see Eriksen, 1995, for a review) to the investigation of basic processes in reading. The flankers task had already been used to study attentional influences on single letter identification, but Dare and Shillcock made the crucial initiative to move up one level – to word recognition. They referred to the new paradigm as “flanking letters lexical decision”, but I prefer to use the more general term “the reading version of the flankers task” since this covers different reading tasks and different types of flanking stimuli. This paradigm was initially developed by Dare and Shillcock as a new means to reveal so-called “parafoveal-on-foveal” effects during reading – that is, the influence of stimuli located left and right of a fixated word on the processing of that fixated word. Crucially, the entire sequence of flanker and target stimuli was presented very briefly (150 ms) in the Dare and Shillcock study. Their results were in line with their predictions. That is, bigram flankers that were related to the target (e.g., RO ROCK CK) facilitated lexical decisions to central target words compared with unrelated flanker (e.g., PA ROCK TH). Crucially, the same facilitatory effect of flanker relatedness was found when the order of the related bigrams was switched (e.g., CK ROCK RO). Moreover, in a second experiment Dare and Shillcock demonstrated that the same findings could be obtained with eye-movement measures (i.e., gaze duration on the fixated word as a function of surrounding stimuli). In an eye-movement study Angele et al. (2013) found the same pattern as Dare and Shillcock (2013), but interpreted their findings as reflecting non-linguistic feature integration, and therefore concluded that these findings were irrelevant with respect to the parafoveal-on-foveal debate.

**Figure 2:**
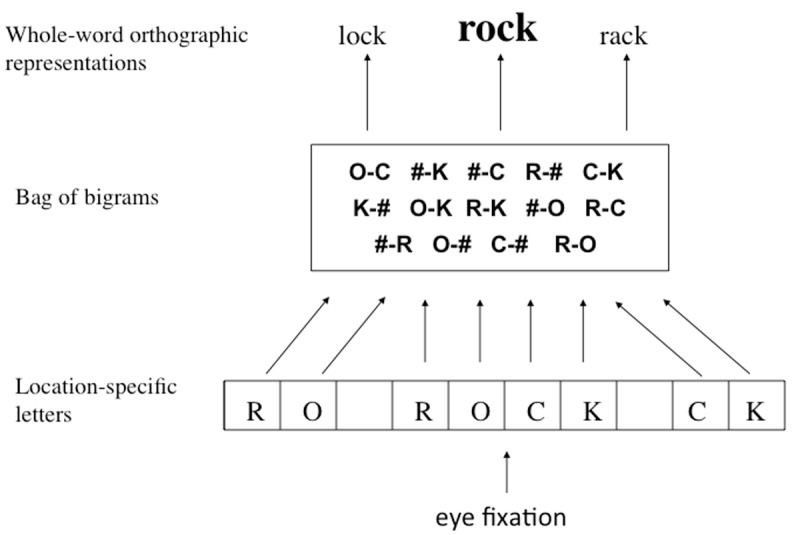
Extension of Grainger and van Heuven’s (2004) model of orthographic processing to the case of multiple words (letter strings separated by spaces). Location-specific letter detectors operate in parallel across multiple words, signaling the evidence that a given letter identity or inter-word space is present at a given location relative to eye fixation. This information is used to activate ordered pairs of contiguous and non-contiguous character combinations (26 letters augmented with the space character—#) stored as an unordered set of open-bigrams (a bag of bigrams). Bigrams then activate whole-word orthographic representations for unique word identification.

My own reaction to the findings of Dare and Shillcock was to propose a theoretical framework that could explain these findings in terms of the spatial integration of orthographic information across the fixated word and orthographic stimuli located left and right of that word. This framework was described in Grainger et al. (2014) and was to form the basis of further theoretical developments (e.g., Snell et al., 2017; Snell et al., 2018), and much discussion (see Snell & Grainger, 2019, and accompanying commentaries), in the field of reading research. The framework is shown in Figure 2. Nevertheless, the exact nature of the mechanisms driving such flanking-letters effects is still under debate. But before further discussing potential underlying mechanisms, what are the facts so far?

At a purely empirical level of analysis, it is now well-established that flanking letters impact on the processing of central target words in the reading version of the flankers task. Shown in Figure 2, we interpreted the initial findings of Dare and Shillcock (2013) as reflecting the spatial integration of orthographic information spanning multiple spatially distinct letter strings (i.e., separated by a space). However, contrary to this interpretation of “orthographic” parafoveal-on-foveal effects, Angele et al. (2013) proposed that they reflect the spatial integration of pre-orthographic visual features. The evidence obtained combining flanker effects with EEG recordings (Snell et al., 2023) nevertheless clearly indicates that the effects are at minima orthographic effects (i.e., involving the processing of linguistic information and not visual information) given that they start to emerge in a time-window, the N250 ERP component, that has systematically been associated with prelexical orthographic and phonological processing (see Holcomb & Grainger, 2006; and Grainger & Holcomb, 2009, for a review). Over and above the operation of spatial integration mechanisms, whether or not these “orthographic” flanker effects reflect, to some extent, open-bigram or more generally open n-gram coding,^7^ remains to be seen (e.g., Snell et al., 2023; Snell, 2024).

A number of additional facts are now well-established. First and foremost, the integration of non-lexical information across spatially distinct adjacent letter strings is essentially driven by orthographic information with no role for phonological information (Cauchi et al., 2020).

When flanking stimuli are real words, on the other hand, then a number of additional effects emerge. These include the impact of syntax, semantics, and morphology on flanker effects. Syntactic flanker effects were first demonstrated by Snell et al. (2017) in a study where the syntactic category (noun vs. verb) of flankers and targets was manipulated. Target and flanker words were otherwise unrelated, yet the syntactic compatibility of flankers facilitated syntactic decisions to target words (i.e., responding that the target was a noun was easier when the flankers were also nouns). Semantic flanker effects were demonstrated by Snell, Declerck, et al. (2018) in a study where French-English bilinguals classified central targets as being an English word or not. Flankers could be non-cognate translations of targets (e.g., loup wolf loup) or unrelated French words (e.g., loge wolf loge). Flanker words that were translations of central target words facilitated lexical decisions to those words. Morphological influences on flanker effects (in French) were first reported in Grainger et al. (2021) and replicated by Beyersmann and Grainger (2024). Target words could be morphologically complex words such as “farmer”, pseudo-complex words such as “corner” (where the pseudo-suffix ending “er” does not function as a suffix – a corner is not someone who corns), and simplex words such as “cashew” (where the ending, following the embedded word “cash” is not a suffix). Flankers were either the embedded word aligned with the beginning of the target word (“farm”, “corn”, and “cash” for the examples given) or unrelated words (e.g., book farmer book). Facilitatory effects of flanker relatedness were found to be significantly greater when related flankers had a transparent morphological relationship with targets (e.g., farm farmer farm) compared with both the pseudo-morphological condition (e.g., corn corner corn) and the non-morphological condition (e.g., cash cashew cash). Grainger et al. (2021) provided a tentative explanation for this pattern of morphological flanker effects and why they diverge from masked priming effects where pseudo-morphological primes (e.g., corner-corn) typically align with truly morphological primes (e.g., farmer-farm), and show greater priming than non-morphological primes (e.g., cashew-cash: see Diependaele et al., 2012, for a review of the evidence). Grainger et al. argued that in masked priming, with primes occupying the same central location as targets, then competition between words for the same location cancels the benefits of a transparent morphological relatedness between primes and targets. That is, in masked priming, processing of the prime would be integrated with processing of the target given the very brief prime duration and the superposition of prime and target stimuli. This would not arise in the flankers task because target and flanker stimuli occupy distinct spatial locations.

Another means of demonstrating context effects on single word processing was provided by Snell and Grainger (2017). Snell and Grainger adapted the post-cued letter-in-string identification paradigm to post-cued identification of words embedded in a sequence of words. In this paradigm, participants are shown a briefly (200 ms) presented sequence of words (e.g., the dog can fly) which are subsequently masked with a post-cue indicating the word to be reported. So, after briefly seeing “the dog can fly” participants would then be presented with “### ### ### ###” and would be requested to report the word that was present at the cued (underlined) location. Performance in this condition is compared to performance in an ungrammatical re-ordering of the same words, with the target word (“dog” in this example) at the same position (e.g. fly dog the can). Results showed superior report of target words when embedded in a grammatical sequence compared with the ungrammatical condition.

The sentence superiority effect has since been replicated in several studies that provide additional insights into the underlying mechanisms. First of all, Wen et al. (2019) recorded EEG in an experiment similar to the behavioral study of Snell and Grainger (2017). The key finding here was that the electrophysiological manifestation of the sentence superiority effect emerged at around 270 ms post-sequence onset. This was taken as evidence that the effect did not reflect slow inferential processes (e.g., I’ve seen the word “the” followed by the letter “d” at the beginning of a 3-letter word and I therefore guess that this could be the word “dog”). The fast-acting nature of the phenomenon is more likely to reflect the rapid activation of a primitive (or “good-enough”, Ferreira & Lowder, 2016) sentence-level representation that then provides feedback to on-going word identification processes (see Dufau et al., 2024, for a further demonstration of early effects of sentence superiority plus a tentative neural localization of such effects).

Declerck et al. (2020) provided further behavioral evidence in favor of the cascaded, interactive-activation account of the sentence superiority effect, while providing crucial information relative to the nature of the primitive sentence-level representation that lies at the heart of this phenomenon. They did so by testing French-English bilinguals with word sequences such as “ses feet sont big” (i.e., a mix of the English sentence “his feet are big” and the French sentence “ses pieds sont grands”). Declerck et al. found a “bilingual” sentence superiority effect and suggested that it reflected the construction of a sequence of language-independent syntactic categories (e.g., pronoun, noun, verb, adjective) that are rapidly activated upon presentation of a sequence of words. The grammaticality of this sequence (i.e., the correct ordering of syntactic categories) then determines the feedback to on-going word identification processes.

An additional role for semantics in driving the sentence superiority effect was demonstrated in the work of Massol and Grainger (2021). That study compared the magnitude of the sentence superiority effect obtained with semantically “normal” sentences such as the “that dog did bark”, compared with semantically anomalous but grammatically correct word sequences such as the “that dog has wings”. In line with the results of Declerck et al. (2020), the Massol and Grainger study found that the sentence superiority effect was principally driven by syntactic constraints (i.e., difference between syntactically correct sequences vs. ungrammatical sequences) but “semantics” was found to contribute about 20% to the overall magnitude of the effect. The extent to which such “semantic” influences on sentence superiority reflect differences in predictability (as measured, for example, by cloze probability) or semantic associations between words in the sequence remains to be seen.

In the final part of this section, I will report the results obtained with more global measures of sentence processing, as opposed to the effects of surrounding context on single word identification as summarized in the preceding paragraphs. Linguists have often used “well-formedness” decisions when testing different syntactic theories. In this task, participants are asked to judge whether a sequence of words is grammatically correct/well-formed or not, under no time pressure. Crucially, in certain conditions, participants are requested to judge the grammaticality of a sequence of words independently of its semantic interpretability (e.g., Chomsky’s famous example “colorless green ideas sleep furiously” should be judged as being grammatically correct). Leaving aside the issue of semantic interpretability and inspired by the success of the lexical decision task in investigations of single word reading, we decided to use a speeded version of well-formedness judgements that we refer to as the “grammatical decision task”: decide as rapidly and as accurately as possible if a given sequence of words is grammatically correct or not.

One phenomenon in particular has emerged thanks to the use of the grammatical decision task. This phenomenon was inspired by results obtained with the lexical decision task and manipulating the order of letters in words or nonwords (i.e., the transposed-letter effects discussed in section 3). Mirault et al. (2018) compared performance to two types of ungrammatical sequence when making speeded grammatical decisions (with the ungrammatical sequences randomly mixed with an equal number of grammatically correct sequences): 1) sequences that were rendered ungrammatical by transposing two adjacent words in a correct sentence (e.g., “the white was cat big” derived from “the white cat was big” by transposing the words “cat” and “was”) – the transposed-word condition; 2) by transposing the same two words embedded in an ungrammatical base sequence (e.g., “the white was cat slowly” derived from “the white cat was slowly”) – the control condition.^8^

Mirault et al. found that “ungrammatical” decisions were harder to make (longer RTs and more errors) in the transposed-word condition compared with the control condition – a transposed-word effect. This is the word-sentence analog of transposed-letter effects.

Mirault et al. (2018) concluded that these transposed-word effects must reflect a certain amount of parallel word processing during sentence reading combined with the influence of top-down syntactic constraints on word-order encoding. Snell and Grainger (2019) further suggested that transposed-word effects might be partly driven by noisy bottom-up position noise, the sentence level analog of letter position noise as implemented, for example, in Gomez et al.’s (2008) “overlap” model of orthographic processing (see Ratcliff, 1981, for an earlier description of this mechanism). Whether or not transposed-word effects reflect partly parallel word processing vs. strictly sequential word processing, remains, however, an open debate. According to the sequential account, transposed-word effects reflect inferential processes (e.g., Gibson et al., 2013; Levy, 2008) operating once serial word identification processes are complete (e.g., Huang & Staub, 2023). This reasoning finds support in studies that have revealed transposed-word effects in error rates but not in RTs (e.g., Huang & Staub, 2023; Liu et al., 2022). Nevertheless, it should be noted that transposed-word effects are systematically stronger under parallel compared with serial presentation of words (see Snell & Melo, 2024, for a summary of the evidence and conclusive findings).

However, our own research (Mirault et al., 2023) suggests that it might be the atypical format of text presentation in RSVP (i.e., serial vs. parallel presentation of text) that is the culprit. In line with this account, transposed-word effects have been found in both RTs and error rates in the auditory version of the grammatical decision task (Dufour et al., 2022) where sequential word presentation is imposed by the modality of presentation rather than by the particular procedure that is adopted for visual presentation (RSVP vs. RPVP).

In a key study, Wen et al. (2021) compared processing of transposed-word and control ungrammatical sequences in an EEG study (Experiment 2 of that study). In line with the results obtained with the sentence superiority effect (Wen et al., 2019, reported above), transposed-word effects emerged in an early-onsetting N400 component with an initial peak around 300 ms post-sequence onset (see also Pegado et al., 2021, for a similar pattern obtained with the same-different matching task). Given that in strictly serial models of eye movements and reading (e.g., EZ-reader: Reichle et al., 1998) each word takes at least 150 ms to process (approximately 150 ms fixation on the word plus the time it takes to move to the next word) one would not expect to see such an early onsetting transposed-word effect. At best (when considering the impact of parafoveal processing – i.e., processing of word N+1 while still processing word N) the EEG could signal a syntactic anomaly when reading the word at position 3 (in 4-word sequences), but that would be identical for the transposed-word and control sequences (see Snell et al., 2023, for evidence for parafoveal-on-foveal effects using fixation-related potentials, Dufau et al., 2024, for a study of sentence superiority using MEG, and Seijdel et al., 2024, for further evidence for parafoveal-on-foveal effects on the N400 component that can be accounted for by lexical activity in OB1-reader).

Another exciting development in the field of written sentence processing is the use of fast-priming as a technique to reveal basic mechanisms involved in deciding if a sequence of words is grammatically correct or not. In Mirault et al. (2022) we combined fast priming with the grammatical decision task imitating the analog of this successful combination for studying visual word recognition (i.e., masked priming and lexical decision – Forster & Davis, 1984). Primes and targets were sequences of 5 words, with each 5-word sequence presented in parallel and the target sequence following the prime sequence after a 100 ms delay. Prime sequences were on screen for 200 ms. We found two sentence priming effects: 1) a simple repetition priming effect (i.e., the prime sequence is the same as the target sequence or not); 2) a transposed-word priming effect where the prime sequence could be formed by transposing two words in the target sequence or by substituting the same words with different words (e.g., “John likes big the car”/“John likes red his car” derived from the target sentence “John likes the big car”). We concluded that the combination of priming (with relatively short prime durations for 5-word sequences) and the grammatical decision task offers much promise for future investigations of the basic sentence-level processes, following letter and word-level processing, involved in reading sentences.

In sum, much of my recent work on sentence processing (in collaboration with numerous colleagues) has mimicked the paradigms, the results, and the interpretation of these results obtained in the field of single word recognition. Section 5 will elaborate on this central point.

## 5. A unifying framework for letter, word, and sentence reading

**Figure 3:**
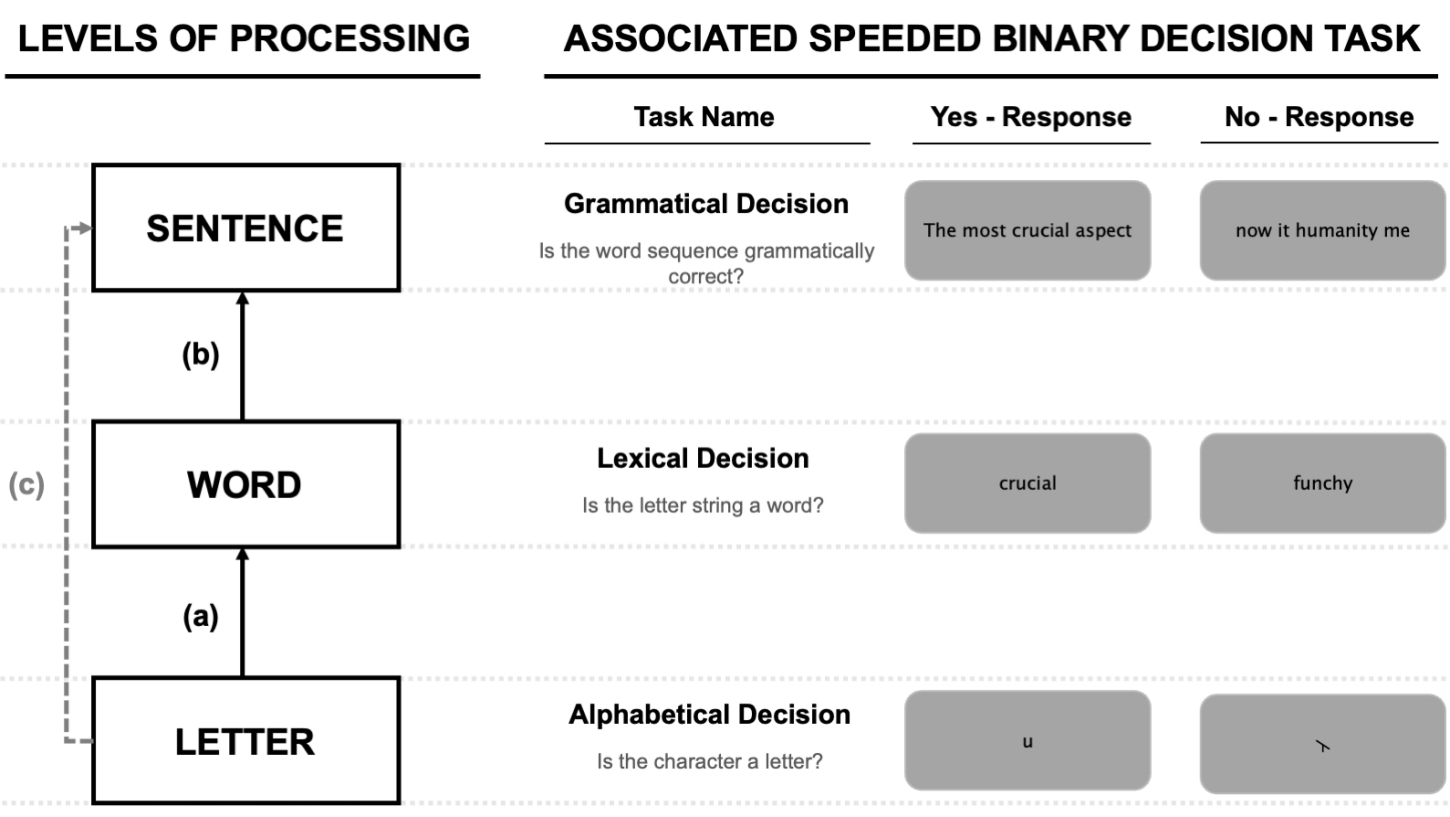
Connecting processing at the letter, word, and sentence levels during reading by using comparable paradigms to investigate processing at each level (Figure from Brossette et al., 2022).

In the previous sections I have pointed to some methodological and theoretical bridges that connect research on letter, word, and sentence processing (see Scaltritti et al., 2021, for a study relating letter identification processes with word identification). These bridges were summarized in Brossette et al. (2022) and I will re-iterate them there. The originality of the Brossette et al. study was that participants were tested in three comparable speeded binary decision tasks designed to reflect processing at three key levels involved in reading: alphabetic decision for individual letter processing, lexical decision for single word processing, and grammatical decision for the processing of phrases and sentences. The main aim of that study was to examine correlations in processing across the three levels. According to the notion of hierarchical cascaded processed, endorsed in the present work, we expected to see significantly stronger correlations between adjacent levels of processing (i.e., letter-word, word-sentence) than non-adjacent levels (i.e., letter-sentence). The logic of this study and the resulting predictions are summarized in Figure 3.

The results of the Brossette et al. study confirmed our predictions. Correlations in standardized RTs were indeed significant when comparing adjacent levels (i.e., letter-word; word-sentence) and non-significant when comparing non-adjacent levels (i.e., letter-sentence). Crucially, this pattern was obtained when partially out the RTs obtained in a non-reading task (speeded semantic decisions to simple line drawings – animal vs. non-animal) which was designed to capture generic speeded binary decision making. Furthermore, an analysis of the RT distributions obtained in the three main tasks (alphabetic decision, lexical decision, grammatical decision) revealed an increase in spread as task complexity increased. This change in the shape of RT distributions was successfully modeled by changing the slope parameter in a simple random walk model (with no other parameter changes). This parameter change reflects differences in the rate of accumulation of information in the three tasks: as the task gets more complex the rate of information accumulation is slower. In sum, I would argue that very similar mechanisms are involved in making alphabetical,^9^ lexical, and grammatical decisions over and above simple speeded binary decision mechanisms. The similarity would lie in the need to decide between linguistically correct and well-matched linguistically incorrect stimuli, with the only major difference arising in the complexity of the stimuli involved.

**Figure 4:**
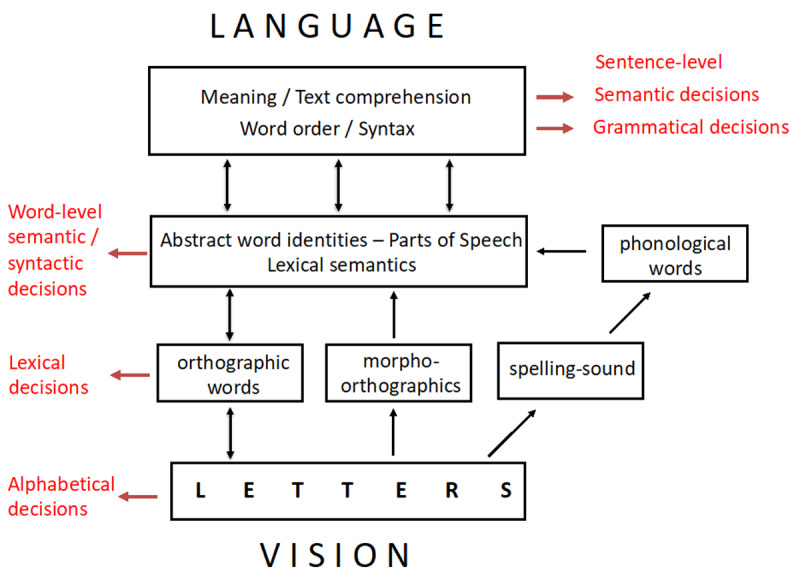
A “box and arrows” representation of the main processing phases involved in transitioning from a visual representation of strings of letters to meaning during skilled adult reading in a language that uses an alphabetic script.^10^ In red are “read-out” mechanisms that connect core processes with decision-level processing required to perform a particular task (Grainger & Jacobs, 1996).

I will end this section by describing a tentative theoretical framework that provides a link between the empirical findings and interpretations of those findings proposed in the preceding sections. The framework is an extension of the one proposed in Grainger (2018; 2022), now including higher-level sentence and text-level processing involved in reading comprehension. The updated framework is shown in Figure 4.

Figure 4 presents an extension of the general framework proposed in my prior work which was focused on letter and word-level processing. Furthermore, this updated framework describes the potential links between core processes at each level (letters, word forms, word-level syntax and semantics, sentence-level processing) and the decision-making mechanisms that are required to perform a task that is thought to tap into a specific level of processing. It is my opinion that computational models of reading need to specify not only the nature of the core representations (e.g., what is an “orthographic word”) but also the decision processes that allow participants to respond in behavioral tasks such as lexical decision. Achieving this level of detail in describing what the literate brain is doing while reading will then facilitate the connection between cognitive and neural processing during reading as well as connecting the binary decision-making tasks that have been the focus of the present essay with more “naturalistic” behavioral measures of text reading such as eye movement recordings. Note that decision processes are generally involved, even with neural measures (EEG, fMRI, MEG: participants are typically asked to do something) and eye-movement measures (decisions about when and where to move the eyes). However, one interesting means of circumventing decision processes would be to measure fixation-related brain activity while participants are simply requested to read a text for meaning (with questions asked once the reading is complete).

## 6. Outstanding questions

Clearly, there is much to be done before we achieve a full understanding of the mechanisms involved in skilled reading. Here, I will simply list what I consider to be some of the remaining issues, and this consideration in by no means intended to be exhaustive (see Snell & Grainger, 2019, for an earlier consideration of such outstanding questions and some tentative responses). Given that the present review article was deliberately focused on my own contributions, it is therefore focused on reading in an alphabetic script with an even more specific focus on studies with the Roman alphabet. At least I am not guilty of being part of the “Anglocentric” approach to reading research quite rightly criticized by David Share (Share, 2008), since most of my research has been in French. Nevertheless, with the aim to develop a “universal” model of reading (e.g., Frost, 2012), one must not only seek general principles that cut-across different levels of processing within a given writing system, such as letters, words, and sentences in an alphabetic system (see Figure 4), but also across different writing systems (e.g., alphabetic, logographic, Braille).

Within the realm of alphabetic writing systems, one interesting comparison is between reading in languages that use the Roman alphabet with reading in Semitic languages. Much research on this particular point has focused on the role of morphology, given the very different ways in which letter-level information is mapped onto morphological information in these two writing systems (see Frost, 2012, for a summary of this work). One key difference is that root morphemes in Semitic writing systems are composed of ordered but not necessarily contiguous letter sequences. I personally take this as clear evidence in favor of a universal process, at least for reading in languages that use an alphabetic writing system, of a process of mapping letters to meaning that does not rely on precise letter locations but does rely on precise letter order information with intervening letters not disrupting the order-encoding mechanism (e.g., A is before C in ABC to the same extent that A is before B). The general principle here could be referred to as “open n-gram coding” (where “gram” here refers to letters). As proposed by Grainger and Ziegler (2011) this coarse-grained mechanism would be complemented with a more precise letter position coding mechanism that would facilitate the association of graphemes and small morphemes (i.e., affixes) with their corresponding sounds and meaning.

As concerns reading in non-alphabetic writing systems, there is a growing body of research on reading in logographic writing systems, principally Chinese, that suggests that Chinese characters act as morphemes that can be individual words, or can combine with other characters to produce words, as is also the case in alphabetic writing systems (e.g., Beyersmann & Grainger, 2023). Although much research has been dedicated to developing our understanding of the role of morphemes in word and sentence reading in different languages, it remains to be clarified how reading words in highly agglutinative languages, such as Finnish, differs from reading sentences in languages that rarely use compounding. One means to evaluate this would be to compare sentence reading with regular between-word spacing and reading text where the spacesbetweenwordshavebeenremoved (see Mirault et al., 2019, for a review of this research).

Finally, there are two cases of exceptional readers, independently of the script that is used, that can provide additional leverage with respect to understanding the universal mechanisms involved in reading. These exceptional cases concern “reading without vision”, or more precisely, reading in severely vision-impaired persons, and “reading without hearing” – that is reading in severely hearing-impaired persons. Research on reading in Braille, a tactile reading system, has highlighted the commonalities between reading through the eyes and reading through the fingers (e.g., Baciero et al., 2022; 2023; Fischer-Baum & Englebretson, 2016; Perea et al., 2015). This research therefore provides encouraging evidence in favor of a modality-independent and script-independent account of skilled reading. As for reading without vision, thanks to my collaboration with Phil Holcomb I have had the opportunity to work on this fascinating topic in collaboration with Karen Emmorey (e.g., Emmorey et al., 2020; Meade et al., 2020; see Holcomb et al., 2024, for a review of this work). As is the case with Braille reading, research on profoundly deaf readers provides another important leverage with respect to revealing the universal mechanisms involved in skilled reading across scripts and modalities.

In sum, one major aim for future reading research is to establish general principles that not only cut across the different levels of processing and different writing systems, but also across reading in different modalities (eyes vs. fingers) and with or without the help of a previously learned spoken language.

## Footnotes

This might seem too obvious, yet apparently it is not obvious to laypersons. I listened to one (non-scientific) celebrity claiming that letters are just arbitrary while words are associated with “things”, therefore we shouldn’t care about letters.

The Reicher-Wheeler task is a data-limited task with a two-alternative forced choice (2AFC) response where the alternative response being compatible with the category of the stimulus being tested (e.g., T vs. C in TABLE, where CABLE is a word). However, much research has shown that a simple post-cued letter identification task (e.g., what was the letter at the 1^st^ position in the stimulus that you just saw?) generates pretty much the same pattern of findings as the 2AFC task, thus providing further evidence against a sophisticated guessing account of word superiority effects.

Upon moving back to his homeland, Cattell became the first Professor of Psychology in the US (University of Pennsylvania), co-founded the journal “Psychological Review”, and was Editor of the journal “Science” for almost half a century.

Note that a more recent model of orthographic processing (Snell, 2024) uses the locations of letters left and right of fixation to infer letter order via contiguous letter combinations.

See Perea et al. (2023) and Romero-Ortells et al. (2024) for further evidence in favor of a distinction between location and order encoding. Perea et al. tested transposed-letter effects with the transposed letters occurring across a line-break. Romero-Ortells et al. presented the critical transposed-letter pseudowords and their controls in a vertical syllable-by-syllable format. Both studies found that transposed-letter effects remained robust when horizontal location information was disrupted, hence providing support for the role of a more abstract order-encoding mechanism in driving transposed-letter effects over and above effects of positional uncertainty.

Note that open n-gram coding includes 1-grams (i.e., single letters) hence implementing the bag-of-letters proposed in Snell, Bertrand, et al. (2018).

Mirault et al. (2018) actually used quadruplets of sequences such that the same set of words were tested in the transposed-word and control conditions (see their Table 1 for the complete design).

Note that I have often used the term “alphabetic” when referring to the “alphabetic decision task” since this is the term that has been used in prior research. However, in order to be consistent across levels of processing I now prefer “alphabetical”.

I would like to recognize the immense contribution of Sally Andrews to this field, particularly well reflected in her edited volume titled “From inkmarks to ideas” (Andrews, 2006), the title of which perfectly summarizes the aims of the present essay. Sally, who sadly left us in 2022, was a truly great scientist and a wonderful person.

## Appendix

List of all my scientific collaborators with whom I have published (up to May 2024). I provide this information so that the “so many” in the Churchill citation can be evaluated. I have not listed the number of co-authored publications, since every single publication counts.

Alario F.-X., Albrand J.-P., Alvarez R.P., Anderson J., Anton J.-L., Aparicio X., Armando M., Avramiea A.E., Baayen R.H., Badier J.-M., Balota D.A., Bastien M., Beauvillain C., Bellocchi S., Bendahman L., Bertrand D., Beyersmann E., Biardeau A., Bijeljac-Babic R., Blackford T., Bolger D., Bonin P., Boros M., Boucart M., Bouttevin S., Broqua F., Brossette B., Brysbaert M., Carrasco-Ortiz H., Carreiras M., Casalis S., Castles A., Cauchi C., Chanceaux M., Chanoine V., Chauncey K., Chen S., Colé P., Coltheart M., Conrad M., Cornelissen P., Courrieu P., Crepaldi D., Dalmaijer E., Dandurand F., Declerck M., Dell’Acqua R., Diependaele K., Dijkstra T., Dimitropoulou M., Ducrot S., Dufau S., Dufour S., Duñabeitia J.A., Eben C., Eddy M., Emmorey K., Fabre L., Fagot J., Farid M., Farioli F., Fernández-López M., Ferrand L., Fléchard L., Fournet C., Frank T., Frenck-Mestre C., Frost R., Gabrieli J., Geyer A., Giraudo H., Glotin H., Goslin J., Granier J.-P., Guerre-Genton A., Hannagan T., Hansen P., Hartsuiker R., Hernández J.A., Holcomb P., Hoshino N., Huestegge L., Isselé J., Ivanova I., Jacobs A.M., Javourey-Drevet L., Job R., Kerr E., Kezilas Y., Kingma B., Kiyonaga K., Klein M., Koch I., Ktori M., Kuperberg G., Lassault J., Lavaur J.-M., Le Goff K., Leflaëc C., Legou T., Leibnitz L., Lemaire P., Lemhöfer K., Lété B., Longcamp M., Lopez D., Madec S., Magnan A., Magnuson J., Mahnich C., Majerus S., Marzouki Y., Massendari D., Massol S., Mathôt S., McGonigal A., Meade G., Meeter M., Méot A., Midgley K., Millman R., Millogo V., Mirault J., Misra M., Mitra P., Montani V., Montant M., Moret-Tatay C., Morris J., Mousikou P., Mulatti C., Muneaux M., Navarrete E., Nazarian B., New B., O’Regan J.K., O’Rourke T., Okano K., Ordonez Magro L., Pallier C., Pattamadilok C., Pech-Georgel C., Peeters D., Pegado F., Perea M., Peressotti F., Petit J.-P., Philipp A., Porter J., Pu H., Quelen F., Radach R., Rastle K., Raymondet P., Reder L., Rey A., Riès S., Roth M., Rousselet G., Runnqvist E., Sandra D., Sasburg O., Scaltritti M., Schoonbaert S., Schriefers H., Schroeder S., Segui J., Serota R.M., Sessa P., Simpson M., Snell J., Spinelli E., Stephan D., Stevens M., Stoianov I., Strijkers K., Szwed M., Taft M., Theeuwes J., Thorpe S., Tosatto L., Touzet C., Truc C., Tydgat I., Van Assche E., Van der Linden L., Van der Stigchel S., Van Kang M., Van Leipsig S., Vandendaele A., van Heuven W., Velay J.-L., Vitu F., Voga M., Warnier P., Welvaert M., Wen Y., Wheat K., Whitney C., Winsler K., Yap M., Yeaton J., Yum Y., Ziegler J.C., Zwitserlood P.
